# End-To-End Computer Vision Framework: An Open-Source Platform for Research and Education †

**DOI:** 10.3390/s21113691

**Published:** 2021-05-26

**Authors:** Ciprian Orhei, Silviu Vert, Muguras Mocofan, Radu Vasiu

**Affiliations:** Department of Communications, Politehnica University of Timișoara, 2, Piata Victoriei, 300006 Timișoara, Romania; ciprian.orhei@cm.upt.ro (C.O.); muguras.mocofan@upt.ro (M.M.); radu.vasiu@upt.ro (R.V.)

**Keywords:** Computer Vision Framework, Computer Vision, pipeline architecture, benchmarking, deep learning, neural networks, reproducible research, machine learning

## Abstract

Computer Vision is a cross-research field with the main purpose of understanding the surrounding environment as closely as possible to human perception. The image processing systems is continuously growing and expanding into more complex systems, usually tailored to the certain needs or applications it may serve. To better serve this purpose, research on the architecture and design of such systems is also important. We present the End-to-End Computer Vision Framework, an open-source solution that aims to support researchers and teachers within the image processing vast field. The framework has incorporated Computer Vision features and Machine Learning models that researchers can use. In the continuous need to add new Computer Vision algorithms for a day-to-day research activity, our proposed framework has an advantage given by the configurable and scalar architecture. Even if the main focus of the framework is on the Computer Vision processing pipeline, the framework offers solutions to incorporate even more complex activities, such as training Machine Learning models. EECVF aims to become a useful tool for learning activities in the Computer Vision field, as it allows the learner and the teacher to handle only the topics at hand, and not the interconnection necessary for visual processing flow.

## 1. Introduction

Computer Vision (CV) is an interdisciplinary field which deals with understanding digital images or videos, as well or even better than humans do. The main tasks of CV are acquiring, processing, analyzing, and understanding the environment through digital images [1]. Some high-level problems which are successfully tackled by CV are optical character recognition (OCR) [2,3,4], machine vision inspection [5,6], 3D model building (photogrammetry) [7,8], medical imaging [9,10], automotive safety [11], motion capture, surveillance [12], fingerprint recognition [13,14], face recognition, and gesture recognition [15,16,17].

**Figure 1:**
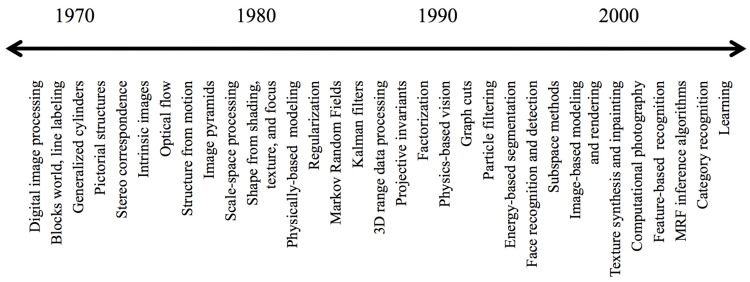
Evolution of computer vision [17].

As the CV topics evolved over the time (see Figure 1), the complexity of the required architectures did too. Pipelines can include image acquisition from image sensors; preprocessing to enhance the image such as reducing noise; selection of region of interest such as background subtraction; feature extraction that would reveal lines, edges, shapes, and textures; high-level processing relevant to the application; and finally, decision-making such as classifying an object as being a car [1,18,19].

Complex open research topics from the CV domain are solved nowadays using Machine Learning (ML), Neural Networks (NN), or Deep Learning (DL) techniques. These techniques solve problems by learning patterns from a huge amount of data and retaining the gained knowledge into model structures and weighted parameters with the aim of solving trivial or corner case challenges [20,21].

**Figure 2:**
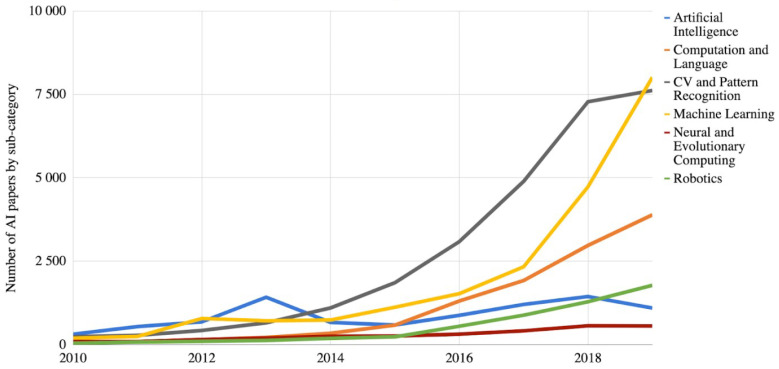
Number of AI papers on arXiv, 2010–2019 [22].

This natural blend of Artificial Intelligence (AI) in solving complex CV topics is evidenced in the evolution of increased number of AI papers on arXiv, overall, and in a number of subcategories. In Figure 2, the graph shows the number of AI papers on arXiv by each paper’s primary subcategory, and we can observe that the increase is constant in the AI domain and CV domain.

**Figure 3:**
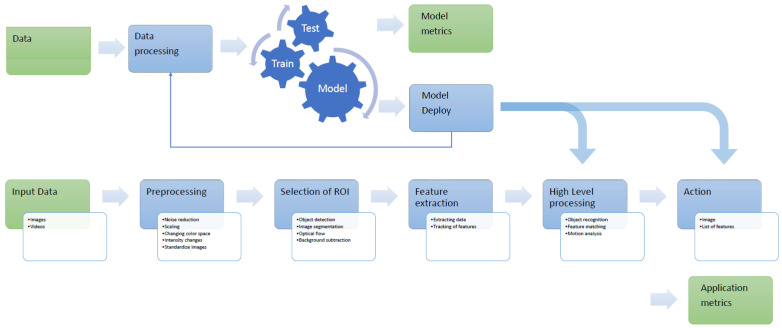
Framework of a modern CV pipeline.

Considering the long-term evolution of CV systems and increasing number of applications, multiple implementation solutions were used to obtain the desired pipelines. Optimization of the CV system may vary by system and considers multiple parameters in doing so. A low-power such system for mobile phones may have a limited GPU and CPU SIMD instruction set which will cause a thinning of the pipeline. However, in any case, the modern CV pipeline is a blended system that include AI elements, such as ML or DL, and classical image processing activities, as we can see in Figure 3.

Even if the programming language is just a tool for implementing the means of the image processing algorithm, choosing the best fitted one is not a trivial task. Usually this choice is dictated by environment conditions, such as the hardware in which the application will run, the purpose of the application, the sensors it needs to interact with and so on.

We propose a Python-based CV framework, the End-to-End CV Framework (EECVF) (https://www.cm.upt.ro/projects/eecvf, accessed on 22 April 2021 ) [19,23], whose purpose is to run custom pipelines designed by the user. The open-source framework can be found in the GitHub repository https://github.com/CipiOrhei/eecvf (accessed on 22 April 2021), under the MIT License.

EECVF provides users with built-in CV algorithms for image manipulation, image smoothing, edge detection, line detection, and semantic segmentation. Even if certain jobs are provided by default, users will discover that adding new features is a facile task in our proposed framework. The provided features are developed using Python based open-source libraries. To be able to respect the current CV pipeline requirements, the framework offers ML models for edge detection and semantic segmentation that users can retrain as needed. The framework has a separate block that handles AI activities, in which users can find the existing models or add their on. We use the generic AI term because, from an architectural point of view, the inner details of the model used (NN or ML) are not visible outside (to the user or to other blocks).

The term “End-to-End” points out the ability of the framework to execute multiple steps of a CV process as a one-click solution. The framework has the capability to handle the complete cycle of create–train–evaluate of an AI model, to run a CV application using the model, and in the end, to evaluate the results and plot them without any user intervention. The assumption of EECVF is that tuning the model, running the CV application, and evaluating the results do not run concurrently. All these steps are accompanied by the debug information that is requested by the user.

EECVF has proven to be a useful software tool for day-to-day research and teaching activities. Using EECVF will shift the attention from developing and maintaining the interaction between software components, pipeline manipulation, and tooling to the CV research itself. The modularity and scalability of the framework will facilitate the continuous development of concepts that we consider will keep EECVF up-to-date in the ever changing CV world.

This work is a extension of our presentation of the EECVF from in [19] and it is structured as follows. In Section 2, we present similar frameworks that were found in the scientific literature and a comparison between them. In Section 3, we describe our proposed framework, EECVF, followed by a use case example in Section 4. In Section 5, we will present the benefits of using this framework in learning activities, and in Section 6, we draw conclusions upon our work.

## 2. Related Work

In this section, we present similar frameworks that we found in the research literature and a comparison is summarized in Table 1. Our analysis highlights the main points of each entry, respective, and the year and programming language they were development in.

The presented state-of-the-art review focuses mainly on frameworks that can handle more generic applications of the CV pipeline, even if they are focused only on the ML or DL part. We excluded frameworks that only focus on one use-case to solve or handle a certain architecture.

All the frameworks described in this section, even our EECVF, use external image processing toolboxes or libraries but these are two different aspects from our point of view. Libraries are useful resources and have been developed over time on several programming languages but they focus on solutions for certain problems and less on the overall system view. Frameworks, compared to libraries, should concern more with the big picture of the CV pipeline flow. Example of popular libraries are MatWorks [24] (for Matlab or C/C++), OpenCV [25] (for Python, Java or C++), CImg [26] (for C++), MatlabFns [27] (for Matlab or Octave), and OpenAI [28] (for Python).

**Table 1:** Comparison of different CV frameworks.

Framework	Year	Language	Main Points
WEKA [29]	1994	C	Focused on ML and Data Mining algorithms
		C++	Open source
			GUI for developing ML data flows
WEKA3 [30]	2009	Java	Focused on ML and Data Mining algorithms
			Open source
			GUI for developing ML data flows
Rattle [31,32]	2011	R	Focused on ML and Data Mining algorithms
			Open source
			GUI for developing ML data flows
DARWIN [33]	2012	C++	Open source
			CV and ML algorithms
			Python wrapper
			GUI for developing ML data flows
FIJI [34]	2012	Java	Open source
			CV algorithms
			Biological-image analysis specific
			Plug-in to Matlab, ITK and WEKA
eMZed [35]	2013	Python	Open source
			Specialized for chromatography (LC)/MS data analysis
			GUI for non-programmer users
AVS [36]	2015	C/C++	Open source
			CV algorithms with clear presentation for teaching
			GUI for non-programmer users
HetroCV [37]	2016	C/C++	Programmer-directed auto-tuning framework
			CV and ML algorithms on CPU-MIC platform
			Focused on run-time on HW optimizations
DeepDiva [38]	2018	Python	Accessible as Web Service through DIVAServices
			Open source
			DL and ML algorithms
Chainer [39]	2019	Python	Deep Learning Framework for Accelerating the Research Cycle
			Open source
			CV and ML algorithms

Waikato Environment for Knowledge Analysis (WEKA) is not a single program, but rather a set of tools bound together by a common user interface. For designing the interfaces, they have taken the view that a tool will ultimately reside alongside other end-user applications such as spreadsheets, word processors, and databases [29]. The WEKA original framework was developed in C and C++ but the latest version WEKA3 is developed in Java now used in many different application areas, in particular for educational purposes and research [30].

The R Analytical Tool To Learn Easily (RATTLE) is a graphical data mining application written in R language. The main goal of this framework is to ease the transition from basic data mining to sophisticated data analyses using a powerful statistical language. Rattle’s user interface provides an entree into the power of R as a data mining tool [31,32].

The DARWIN framework is twofold system based on C++ programming language that aims to provide infrastructure for students and researchers to experiment with and extend state-of-the-art methods. They provide infrastructure for data management, logging and configuration with a consistent and well documented interface for machine learning practitioners [33].

Fiji is an open-source software framework that focuses on image analysis in biology. The framework combines powerful software libraries with a range of scripting languages to enable rapid prototyping of image processing algorithms. Fiji is a distribution of the popular open-source software ImageJ [34].

The eMZed is Python-based framework tailored for mass spectrometry users who want to create tailored workflows for liquid chromatography and data analysis. The framework specifically addresses non-expert programmers with the goal to establish a comprehensive list of basic functionalities [35].

Adaptive Vision Studio (AVS) is a software tool for creating image processing and analysis algorithms. This framework has been tested on a post-graduate computer vision course from Automatic Control and Biotechnology at Silesian University of Technology. AVS has proven to be a powerful environment with ready-for-use image analysis filters for computer vision experts and beginners [36].

HetroCV is an auto-tuning framework and run time for image processing applications on heterogeneous CPU-MIC platforms. In HetroCV, the image processing pipelines is composed by computation units like Map, Stencil, and MapReduce. The main benefit of this framework is the fact that it uses program statistics extracted from the computation units to predict the optimal tuning parameters on-line [37].

The DeepDIVA framework is designed to enable a quick and intuitive setup for experiments that should be reproduced for analysis. The framework offers functions to keeping track of experiments, hyperparameter optimization, and visualization of data and results [38].

Chainer framework is a flexible, intuitive, and high-performance means of implementing the full range of deep learning models for researchers and practitioners. The framework provides acceleration using GPU with familiar Python libraries [39].

From our literature review, we can conclude that no framework specifically handles the full pipeline. For example, we can imagine a use case where we desire to train a specific edge detection ML model, use it inside a CV application together with a classical edge detection algorithm and evaluate the edge results at the end.

Furthermore, most of the presented frameworks focus more on the ML algorithms and provide tools to understand the inner workings of the models. Chainer [39] even goes one step forward and focuses on the way one can optimize the ML pipeline on an specific accelerator.

An interesting aspect is the fact that recent framework solutions have chosen Python as the main programming language. The main benefit for this selection is the facile interaction with the OS environment (Linux or Windows) and the fact that a considerable amount of libraries exist with ML, DL, or CV algorithms.

## 3. Proposed CV System

EECVF is an easy to use, modular, and flexible framework designed for researching and testing CV concepts. The framework does not require the user to handle the interconnections throughout the system. The users do not need to concern themselves with the strategies they need to use for transferring data (input or processed one) from a job to the next or from a block to another (e.g., Applicationblock to Benchmarkblock). We consider the framework easy to use because the system offers the user jobs and services to configure the desired pipeline.

EECVF is constructed in a modular programming software design fashion, with all functional components being independent. This was a relevant aspect when we constructed the framework, as we desired to allow the users to use just one block or several blocks in their desired activities.

All the components, high-level blocks or low-level jobs, respect the concept of data coupling. We aimed to have a loose coupling between software elements so the dependencies would be minimal and the data flow slim. This aspect of construction helped the functionality of the EECVF to be scalable. New features, job or service, can be easily integrated in the framework by users, without any need for refactoring, changing or adapting any existing features or concepts inside.

Flexibility of a software system can be defined as how easy it is to reuse or increase the functionality of classes or modules in similar contexts without the need of redesign or modification of existing architecture [40]. The proposed framework is constructed upon this concept. EECVF high-level architecture easily permits adding new jobs in any of the component blocks without the need for modifying the existing modules, except the interfaces of the blocks to expose the new job to other blocks and users. This concept is enforced by the fact that a certain functional block, that represents a job, is used to create new job, without any modification in the used job.

In recent years, Python—an interpreted, high-level programming language—became the de facto standard for exploratory, interactive, and computation-driven scientific research [41]. We chose Python because of its capabilities to interconnect multiple blocks of our environment and smooth switching between operating systems. To make EECVF more facile, the users can just run the setup_framework Python module, which will install all the requested libraries and dependencies.

Another aspect for choosing Python as the main programming language for EECVF is the capability of it to interconnect sensors to the system easily. We considered this aspect as important in the construction of EECVF as we desire to permit sensors to inject input data streams directly in the pipeline. As a naive example user can find the example_main_camera_video module where the pipeline is configured to obtain data directly from a video camera connected to the system.

### 3.1. High-Level View

**Figure 4:**
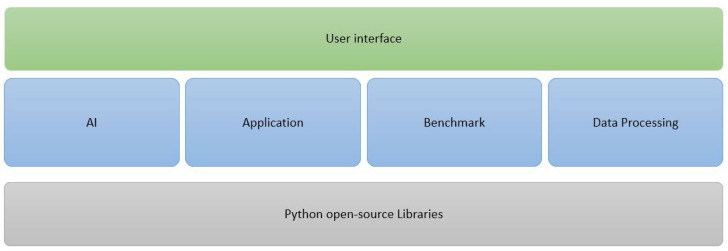
EECVF blocks.

One of the desired outcomes of the framework is to unify different stages of the vast CV research domain. Figure 4 shows the blocks forming EECVF. Treating all the blocks of the pipeline as one component, we reduce the number of redundant operations and calculations by eliminating duplication of data and interfaces throughout the system.

The framework is following a Facade design principle; the Userinterfaceblock acts as a Facade to the entire application. Facade principle states that a complex subsystem should provide an interface which limits interactions with lower layers of architecture, offering a simple channel of communications between user and software functionality [42]. In the proposed framework, the user is not required to interact with the second layer (AI, Application, Benchmark, or DataProcessing) in order to use the application. Every job or service provides a method with the scope of abstracting from the user the necessary sub-system actions needed in a block for execution.

Jobs and services from each block are exposed to the rest of the system via interfaces. This mechanism isolates the inner works of each element from the user and between the elements themselves. As such, the users would only need to focus on the research topic at hand and not on the tools they have to use.

To understand how EECVF inner architecture works, we need to define the terms:

- a job is an action with an added value for the CV pipeline or AI module, typically an output;
- a service is an action that ensures the proper functionality and configuration of the framework;
- a port is defined as a channel for the data that is passed between jobs or blocks of the framework; and
- a wave is a full execution of the pipeline for one frame of the input data.

**Figure 5:**
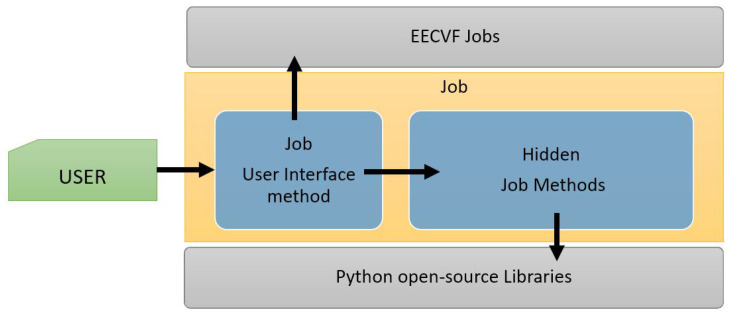
Job structure overview.

Every job offers a public method exposed to the user which can be configured via parameters. This method handles the necessary changes in the system configuration and triggers other necessary jobs from one or several blocks, depending on the nature of the job. As we can see in Figure 5, besides the method for user interface, a job can have multiple private methods that handle the functionality, with or without external python libraries.

Users can opt for only one of the blocks or for several of them. For example, only the Applicationblock to run a simple CV pipeline, or the Applicationblock and the Benchmarkblock to execute and evaluate the results.

### 3.2. AI Block

**Figure 6:**
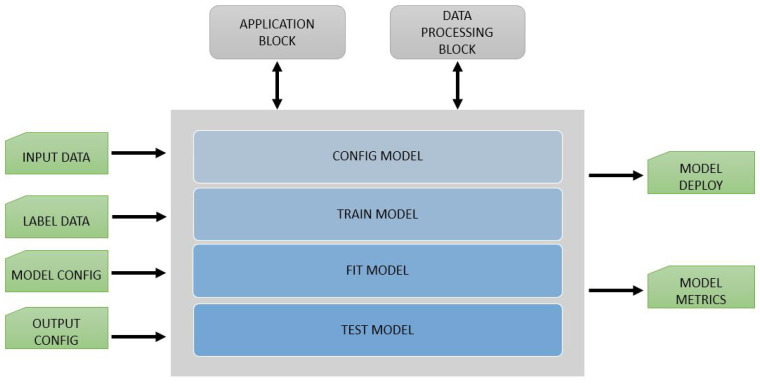
AI block.

This block of the framework handles the training and customization of ML, NN, or DL models. An overview of the block can be seen in Figure 6. With this block, EECVF desires to isolate this specific activities from the CV pipeline. This separation is needed because these are made prior of any usage of the model in any pipeline.

In this block, users can find jobs that will trigger ML semantic segmentation models or edge detection models that can be trained according to desired pipeline needs. Jobs from this block respect the principle presented in Section 3.1, and so the user can easily configure the training using method parameters.

Users can define their own AI models or they can use third-party models already configured in EECVF. Due to the vast model variants and use case variants, it will be the user’s responsibility to define the model and the inner workings of the training process inside the block.

EECVF will provide jobs that will help the interconnection between the AIblock and the other blocks. An example of such an interconnection would be to set up a job in the Applicationblock that uses the model checkpoint output files that were generated after training. Another job that can be exported to a different block of the framework is the validation of models. Validation can be done inside this block or using the Benchmarkblock.

For the augmentation of data we can use the Applicationblock. By using services provided by EECVF, we can set the data exported as input to our jobs in the AIblock. Of course, users can configure the desired jobs in this block to use directly the augmentation function offered by Python libraries but, for a better observation of the augmentation, we use the Applicationblock.

A benefit of EECVF is the fact that it is not dependent on any particular library for this block. Users can integrate models constructed with any library as long as it is Python-based or provides a Python wrapper.

### 3.3. Application Block

**Figure 7:**
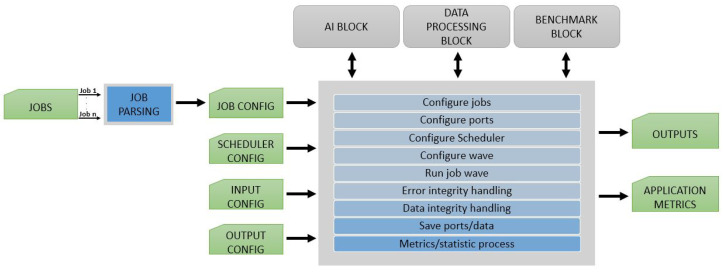
EECVF application block overview.

The Applicationblock handles the CV pipeline in the framework. This block configures the actual order of execution for all triggered jobs and ports that the user describes in the attempt to simulate a use case. The internal design of the Applicationblock is presented in Figure 7. To set up a pipeline, users need to configure jobs with input and output ports, with the input data (images, videos, or any vector data format) and with the schedule for the pipeline.

Like stated before, we constructed the proposed framework to be modular, scalable, and flexible. To enforce this concept, the Applicationblock has processes that attempt to remove duplicate operations, schedule Jobs, and execute algorithms only when needed.

The Proxy design pattern means that a resource is loaded only when it is needed. The Applicationblock follows this approach when executing jobs and services later in the process [42]. This technique allows users to specify what computations they want to make (like do_gaussian_blur_image_job, do_canny_fix_threshold_job, or do_ed_lines_job) and lets the Applicationblock to schedule them after removing duplicates or missing ports, creating a slim pipeline of CV operations. The actual job execution process comes at the final stage of the entire sub-process.

An important intermediate step in the construction of the pipeline is the Jobparsing algorithm. This algorithm avoids duplicate jobs and prepares the system for the scheduling phase. Like we showed in Figure 7, this is an important step prior to running the pipeline. The output of Jobparsing is a JSON file which contains the slimmest list of possible jobs and ports to be used in the pipeline. Another aspect that is determined at this point in time is the priority of jobs. This is done according to the availability of the data as input.

We can observe in Algorithm 1 the inner workings of the job parsing algorithm. The algorithm aim is to sort all the jobs in the pipeline, such as no inputport is missing for a job. Therefore, doing the clustering of processed jobs (the one with input port allocated) and unprocessed jobs (the ones without input port allocated) will result in inactivating any job with missing inputs. The algorithm stops when, during two consecutive iterations, the number of unprocessed jobs does not change. The second part of the algorithm is a verification on the fields (processing level, active, name, input ports, output ports, init function, main function, and so on) of the jobs, so we avoid duplicate execution of them. This logic is valid even for a huge amount of jobs and assures future optimization of the pipelines.

The resulting json file from the job_parsing is the description of the pipeline with each job, input ports, output ports, initialization functions, and run functions.

The initial phase of the Job Parsing algorithm is important because it sets the jobs in an ordered fashion so we can avoid that a job is to be executed before it has the desired ports as input. Next, the algorithm assures that all job that have the necessary inputs can run. The next step is also important because it will eliminate duplicated jobs. Eliminating duplicated jobs does not take in consideration the job name given by the user but the input/output ports and initialization and run function of each job.

**Algorithm 1** Job Parsing Algorithm
  1:Find input job                   ▹ Job with no input, only output ports  2:Process_lvl = 1             ▹jobs which process input data have process_lvl = 0  3:**while**job to process **do**  4:     Cluster processed jobs  5:     Cluster unprocessed jobs  6:     **for** job unprocessed cluster **do**  7:   **if** input_ports of job found in output_ports from processed cluster **then**  8:     Add Process_lvl to job  9:   **end if** 10:     **end for** 11:     Increment Process_lvl 12:
**end while**
 13:Set unprocessed jobs as inactive 14:Sort jobs by Process_lvl ascending 15:**for** Process_lvl in jobs **do** 16:     **if** duplicate job**then**            ▹job name is not considered in this verification 17:   Set job as inactive 18:     **end if** 19:
**end for**
 20:Write active jobs to JSON file


Removal stage done early in the process leads to a Flyweight design approach. Sharing common parts between multiple CV jobs keeps RAM usage as low as possible plus an increase of overall runtime due to a lack of duplicate operations [42]. For instance, when running three jobs (e.g., CV algorithms), each needs to compute a Gaussian Filter, and only one computation of Gaussian algorithm is actually done. Of course, this depends on the level of granularity that the functional implementation offers.

Every job from the Applicationblock has three phases: initialization, run, and termination. The initialization phase for each job is executed once in the beginning of the pipeline. For each wave the run phase of jobs is executed accordingly to the selected scheduler. An important aspect in this step is the mechanism that avoids the duplication of ports, mechanism that becomes more important as the quantity of data increases. At the end of the pipeline, the termination phase is triggered for each job.

In Algorithm 2, we try to describe the flow of a generic Application algorithm. As we can observe, the main function of this block is more focused on the execution environment than on the functionality that is executed. This is an important aspect for the flexibility of this block directly, and for EECVF indirectly.

We can observe from Algorithm 2 that the debug information (e.g., execution time of jobs, debug data, and port listing) is handled for each wave, on one hand, and for the whole CV pipeline, on the other hand. The actual execution of the jobs is configured by the Scheduler that takes in a list of jobs.

**Algorithm 2** Application Algorithm
  1:Start timers, loggers  2:**if**input flow exists **then**  3:    Configure Application accordingly to input source  4:
**end if**
  5:**if**config_json exists **then**  6:    **for** job in job_list **do**  7:     Create job  8:     **for** port of Job **do**  9:      **if** ports not exists **then** 10:         Create port 11:      **end if** 12:      Link Job to Port 13:     **end for** 14:    **end for** 15:    **for** job in job_list **do** 16:     Run Job.Init() 17:    **end for** 18:    **while** wave **do** 19:     Scheduler(job_list) 20:     Save debug data, ports 21:    **end while** 22:    **for** job in job_list **do** 23:     Run Job.Terminate() 24:    **end for** 25:
**end if**



**Figure 8:**
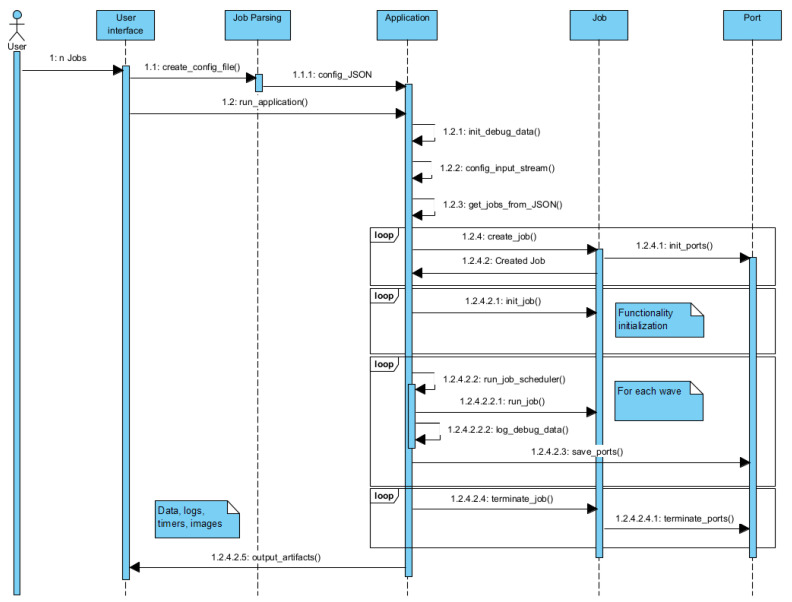
EECVF application block sequence diagram.

In Figure 8, we present the sequence diagram for the execution of CVApplication. As stated before, we can observe that the JobParsing is an important activity in the process, and the fact that the application cannot run without the json file provided by it. We need to differentiate the create_job phase from the init_job phase. Create_job phase refers to the creation of the job object with the equivalent output ports and other inner attributes, while the init_job refers to the initialization of the functionality that the job offers. Init_job and run_job interact with the port every call even if this is not showed in the diagram. The handling of all the ports is done strictly by a port_handle component; this isolation assures the integrity of the data through Applicationblock.

The error handling responsibility inside the Applicationblock is divided between the hidden methods of a job, that should protect against exception that occur in processing, and the block frame together with the scheduler. There are several mechanisms to protect against the execution failing of the Applicationblock. Every port has a validity attribute that is set by jobs when they are filled after processing; if the attribute is false, this will cause that all the jobs that consider the port as input to not execute the current wave. Every job checks, after initialization, that the necessary data and input ports exist; if this is not the case, the job will be eliminated from the pipeline and the depending jobs will not execute. If an error occurs in the run phase of a job, this will be considered corrupted and will be skipped and logged accordingly.

Statistics data from Applicationblock are generated automatically for each wave using DataProcessingBlock. Statistics are generated for each job too accordingly to the nature of it and configuration. The minimum data logged for each job are the output image and run time analysis. Of course the user can configure the quantity of logging data needed for the application.

In a sense, this block can be considered the main block of the framework as CV pipeline is created, customized and executed inside it.

### 3.4. Benchmark Block

The Benchmarkblock handles the evaluation and validation of data from the EECVF. In this block, users can select from several benchmark algorithms to evaluate their results. Typically, this block is used by users after a CV pipeline was executed by the Applicationblock to obtain necessary metrics.

**Figure 9:**
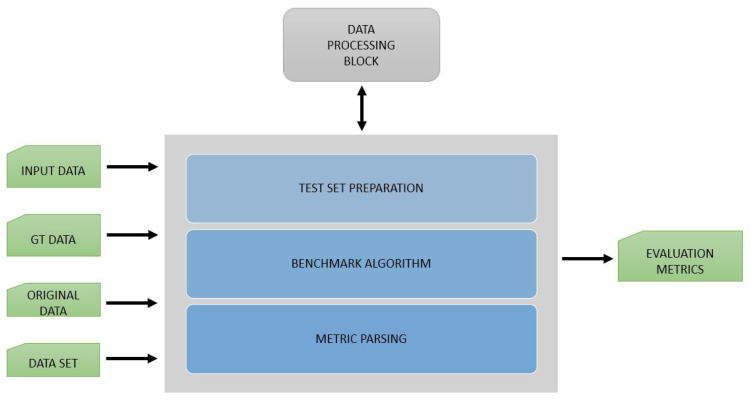
EECVF benchmark block overview.

In our opinion, this block does not need to have a parallel design because, typically, we do not evaluate at the same time that we are running an application pipeline. The block is able to run multiple evaluations for one application. In Figure 9, we present the inner design of the block. The output of the evaluation on multiple sets of data is automatically saved in the results for each set of ports.

A limitation of using this block that we consider mentioning is the fact that the jobs inside this block cannot be integrated with Applicationblockjobs. This limitation exists purposefully because the EECVF environment considers that benchmark is done post-CV pipeline execution.

Using DataProcessingblock, we can visualize and plot the results of the Benchmarkblock for the data we generate using the Applicationblock. Similar to other blocks, the evaluation algorithms that are provided by this block are directly dependent on the domains where EECVF will be used.

### 3.5. Data Processing

The DataProcessingblock is the block that handles the “communication” between users and EECVF. By “communication”, we understand the exchange of data between the framework and the user. This can be exemplified as saved images, tables, plots, or other metrics.

Users can select one of several data manipulation services that can create plots or metrics from the statistics saved by the other blocks. By default, a limited series of data is saved by the EECVF, and they can be found in the Logs folder. The DataProcessingblock has the scope of helping the interactions between users and EECVF and it is used in all the other blocks of the system.

Even if this block does not have an internal design structure, we consider it to be one of the most important ones because all the rest of the blocks depend heavily on it. This block handles the logging mechanism, which is an important feature for our framework. Logging is important for a research-based framework which focuses on offering useful information to the users rather than running quick and silent.

### 3.6. Job Adding

Like we stated before, adding new functionalities to the framework is a facile task because of the architecture chosen when we developed it. All functionalities of the EECVF are to be found under the so-called generic term of job. In the following, we will attempt to explain how to add a new job to the Applicationblock. We chose this block because it has a more specific structure needed for jobs so the main execution loop of the pipeline will be able to use it.

All the needed functionality should be encapsulated in one Python module, to respect the modular principle we imposed on the framework. The new job should offer three public methods, so it can link to the framework: a user-interface method, a initialization method and a run method. A template for the public methods of a job is offered in Application/Jobs/job_template.py.

The user interface method should configure the transition between user interface and the Applicationblock and describe the configuration of the job. As a user can see in the template, the method should have a series of mandatory parameters: name of input port with respective wave (we can process an input from a past wave) and level (pyramid level or custom); name for one or several output ports and parameters that are needed for the actual function.

Inside the user interface job method, users should create lists of input and output ports with a specific format. For transforming the port name and size, we offer specific services which one can use for creating the name and size of the port (transform_port_name_lvl and transform_port_size_lvl). Another important aspect that this method handles is to specify the initialization function and run function for the new job and configure the list of parameters that those two functions should receive.

When adding a new job, if users would like to use an existing job, as inner steps of the new functionality, they should configure them in the user interface method. This is recommended because it will help the framework to maintain a slim pipeline and not have different jobs that do the same functionality.

Both init_function and main_function for the new job should handle the integrity verification of the used ports and avoid crashing due to exceptions on run-time. These basic aspects are covered if user respects the offered template for this public methods.

After the three public methods are constructed, the user only needs to add the user interface method to the interface of the Applicationblock (Application/__init__.py) and the new module to the package init module (Application/Jobs/__init__.py).

The adding of new jobs remains the same for other blocks, with the benefit of needing only one public method that represents the functionality interface with EECVF. Another important aspect to take in consideration is that, if external libraries or repositories are used when adding new jobs, dependencies have to be added to the requirements.txt file. This is important for future users to be able to trigger the new jobs.

## 4. Example Use Case

In this section, we will consider an example of an application that will attempt to present the benefits of using EECVF for day-to-day research. We know that research is a methodical activity but not always clean in the incipient phases, a fact that we like to include in our experiment. Because we desire to present the benefits that our framework can bring for research work, we will not focus on fine tuning the models or cleaning up the pipelines for better results. Rather we will complicate the pipeline with paths for presenting more results.

The example that we present in this section shows how to configure the EECVF to train several ML models, how to use the training output into a CV pipeline and how to evaluate the results at the end. All the jobs that are used in this example are available for users by default in the framework. One can reproduce the example by running main_eecvf_jurnal.py from the framework. All the necessary dependencies (libraries, sub-repositories) are installed when running the setup_framework.py module.

We consider an experiment where we like to determine the best edge detector for urban scenario when the images are segmented prior of edge map processing. To do so, we consider two datasets specialized for this scenario: LabelMe Facade [43,44] and TMBuD [45]; two semantic segmentation models: U-Net [46,47] with VGG16 [48] encoder and SegNet [49] with ResNet-50 [50] encoder; and Canny [51], Shen-Castan [52], and Edge Drawing [53] edge detection algorithms.

**Figure 10:**
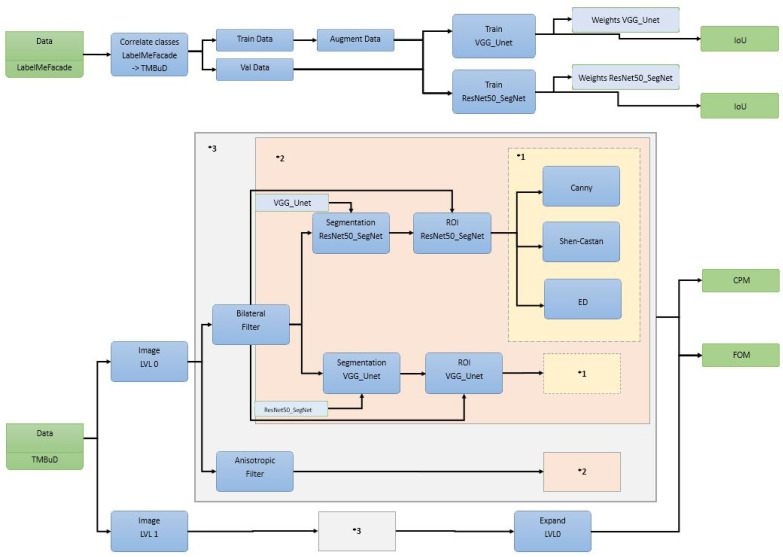
EECVF example logical scheme.

The complete pipeline is presented in Figure 10. For a better visual understanding, we used several annotation like *VGG U-Net* represents the model weights resulting by VGG U-Net training and *ResNet-50 SegNet* represents the weights obtain by ResNet-50 SegNet training; *1 represents the edge detection algorithms used; *2 represents the part of the pipeline in which we apply the segmentation, group the resulting semantic classes into foreground and background, intersect the binary map with the image, and apply the edge detection block algorithms; and *3 represents the block of the pipeline where we apply block *2 on the resulted image after smoothing it with Bilateral Filter [54] and Anisotropic Filter [55]. In the end of the pipeline, we will evaluate using Intersection over Union or Jaccard index (IoU) for the semantic segmentation results and the popular Figure of Merit (FOM) [56] and Correspondence Pixel Metric (CPM) [57] for the edge maps.

The selected complex pipeline, which runs over several pyramid levels, was chosen to highlight the benefits of the framework. As we will see in this section, the user triggers in the final pipeline a number of 752 jobs and saves only 92 from the 902 ports that the application constructs. From our point of view, the fact that a user can describe a pipeline (see Figure 10) and configure which ports to save and which to evaluate demonstrates the fact that the framework is easy to use.

As we can see in Figure 10, we would evaluate the results when the pipeline uses the original image size, Pyramid Level 0, but we would like to see the effects upon the edge map resulted if we process one level lower in the pyramid and reconstruct the edge map back to the original level. Reconstructing features obtained in lower pyramid levels is a common practice in CV domain [58,59].

Another aspect we would like to present in our experiment is the EECVF capability of error handling. To do so, we have set the pipeline to run for Pyramid level 2 even if the do_pyramid_level_down_job has been given the parameter number_of_lvl=1. This will cause the EECVF to discard in the preprocessing part of Application the entire sub-pipeline. With this intentional fault we wish to exemplify the framework’s capability to handle errors. The most important aspect for a software system like this is the capability to handle faults in the system without stopping the execution.

**Figure 11:**
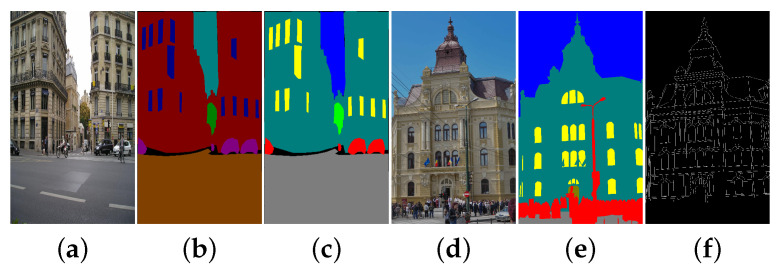
(**a**) Original LabelMe image resized; (**b**) LabelMe original labels; (**c**) LabelMe corelated labels; (**d**) TMBuD original image; (**e**) TMBuD label; (**f**) TMBuD edge ground truth.

In Figure 11, we present the dataset used in our example: LabelMe Facade dataset for training the semantic segmentation models and TMBuD dataset for evaluating the semantic segmentation output and edge maps resulted at the end of the pipeline. Of course, the fact that the two dataset have different perspectives and classes will cause some negative effects on the results but in CV application it is common to use several datasets.

**Figure 12:**
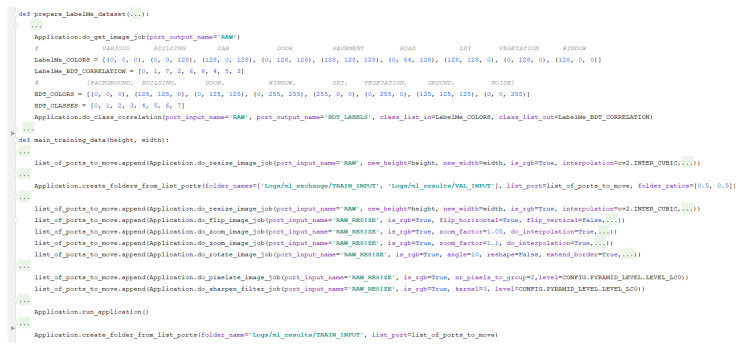
Code snipe of data preparation for training.

**Figure 13:**
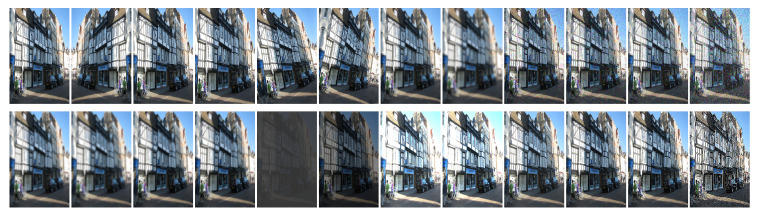
Augmentation done for training.

In Figure 12, we can observe how to set up the augmentation of data for learning of semantic segmentation and how to split the data. We can see in Figure 13 an example of the data augmentation done accordingly to the desired configuration. Using EECVF, we can better observe the effect of the augmentations done to our data, which can be an important benefit when trying to understand the chain of effects.

As stated before, because of the modular implementation of the framework, we can use jobs from one block in other blocks to obtain the desired functionality. In this example, we are using the Applicationblock for data augmentation rather than using the augmentation options offered by Python libraries used for training. We consider that using the EECVF in this way, the researcher better understands the flow of data into the system.

To be able to use the datasets together, we need to correlate the annotation of classes between them. In Figure 12, we can see that the do_class_correlationjob is used for this task. The resulting images are further used in the training process.

We observe in Figure 12 that for preparing the data for the training, we undergo a series of transformations with the scope of enriching the training dataset. We start by changing the resolution of the image to 320 × 520 using the do_resize_image_job. Afterwards, on the resulting resized image, we apply several augmentations like flipping (using do_flip_image_job), zooming (do_zoom_image_job), rotation (do_rotate_image_job), add motion blur (do_motion_blur_filter_job), and so on. To configure the linkage of ports between jobs we just need to add ’RAW_RESIZE’ value to the port_output_name parameter of do_resize_image_job and then use the same value for the port_input_name of the jobs we use for augmentation.

**Figure 14:**
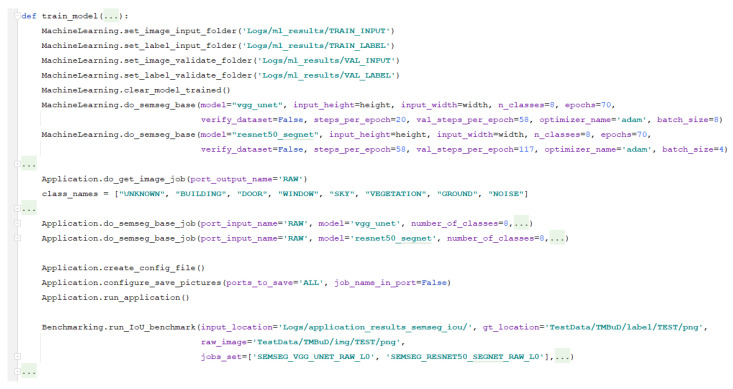
Code snipe of training semantic segmentation models.

In Figure 14, we can see how the VGG-Unet and ResNet-SegNet are configured to be trained. The VGG-Unet is trained with 8 classes, on 70 epochs with a batch size of 8, with 20 steps per epoch on training data and 58 steps for validation data. Similarly, we set up the training of the ResNet-SegNet with 70 epochs with a batch size of 4, 58 steps per epoch on training data, and 117 steps for validation data. Of course, we can fine-tune extensively the networks but, for this example, we consider that it is good enough. The do_semseg_basejob provided has multiple models, already provided by the framework, which can be configured using the model parameter.

Using set_image_input_folder, set_label_input_folder, set_image_validate_folder and set_label_validate_folderservices we configure the framework to use the images outputted in the preparation activities. We use the do_semseg_basejob from Applicationblock to train the networks with the stated configuration. This is a facile configuration as we can change the size of the images we use and add or remove augmentation only from the user-interface block.

Another interesting aspect that we can see from Figure 14 is the fact that, for this phase of the pipeline, we use all the blocks from the framework. This configuration has two benefits: permits the user to have a more detailed control over the activities from this phase and helps us exemplify the interconnections that can be done. Another way to execute this phase is to incorporate the augmentation and validation inside the AIblockjob using methods provided by libraries (Tensorflow, PyTorch).

**Figure 15:**
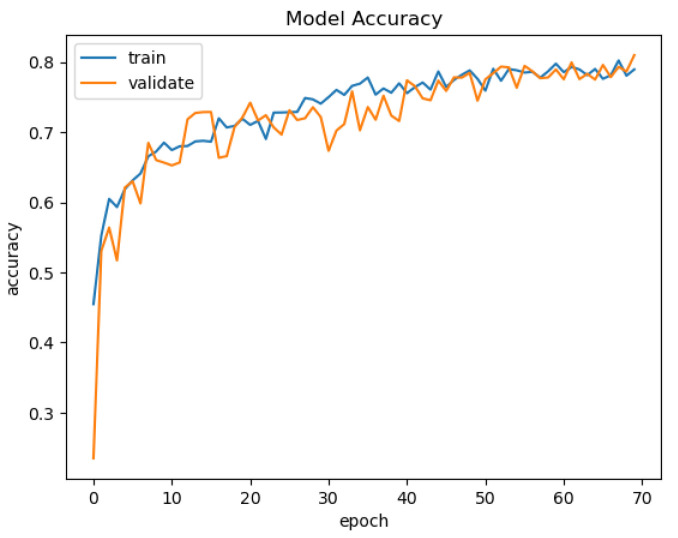
VGG-Unet Acc.

**Figure 16:**
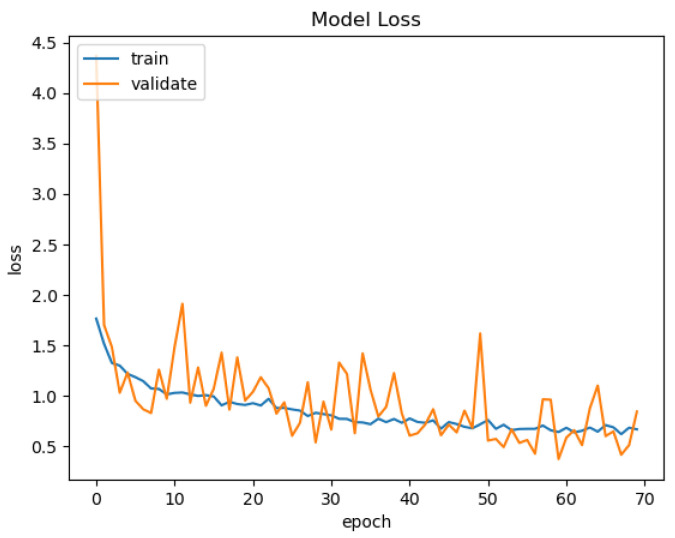
VGG-Unet Loss.

**Figure 17:**
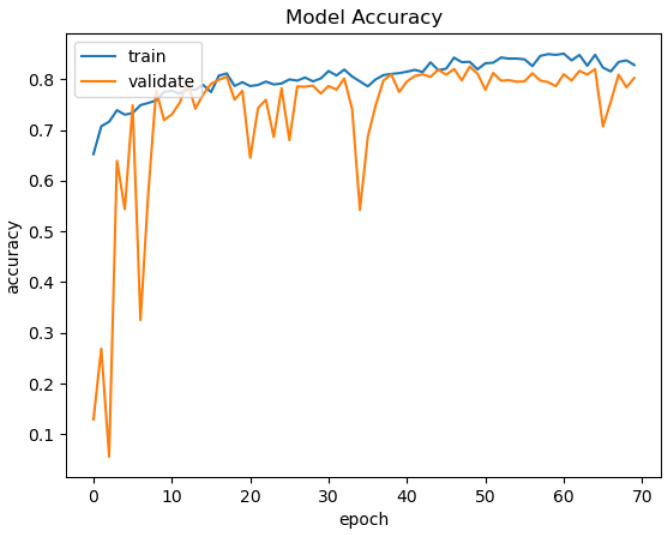
ResNet-SegNet Acc.

**Figure 18:**
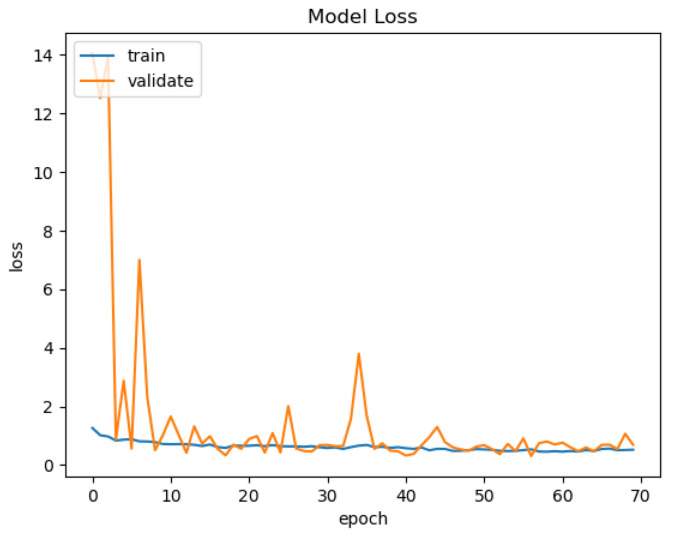
ResNet-SegNet Loss.

In Figure 15, Figure 16, Figure 17 and Figure 18, we present the training results of the models. The corresponding plots are automatically exported by EECVF when doing any training.

**Figure 19:**
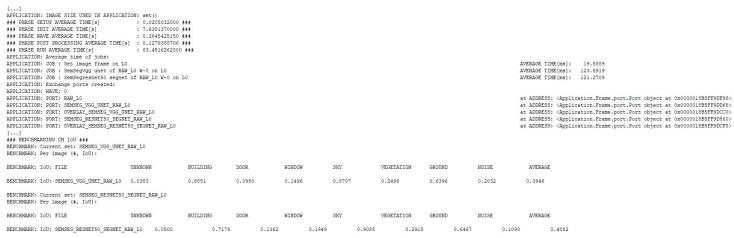
Console output of training evaluation and IoU results.

In Figure 19, we present the results of the evaluation of the models using the IoU metric. Evaluation of the models is done once inside AIblock in the training mechanism, see Figure 15, Figure 16, Figure 17 and Figure 18, and the second evaluation using Applicationblock. Using the run_IoU_benchmarkjob from Benchmarkblock, we configure the evaluation of IoU, see Figure 14, on the data. The data are processed by the Applicationblock using do_semseg_base_job, job that takes the data from the AI_block.

We configured the Applicationblock to handle the augmentation, the AIblock to handle the training and the Benchmarkblock to handle the evaluation of the models. As we can observe in Figure 19, the framework automatically outputs the average time for every phase, wave or job and also the memory address of each created port.

As we stated in the beginning of the section, we will not further fine-tune the networks as it is not in our scope, even if this would be the case for a normal application.

**Figure 20:**
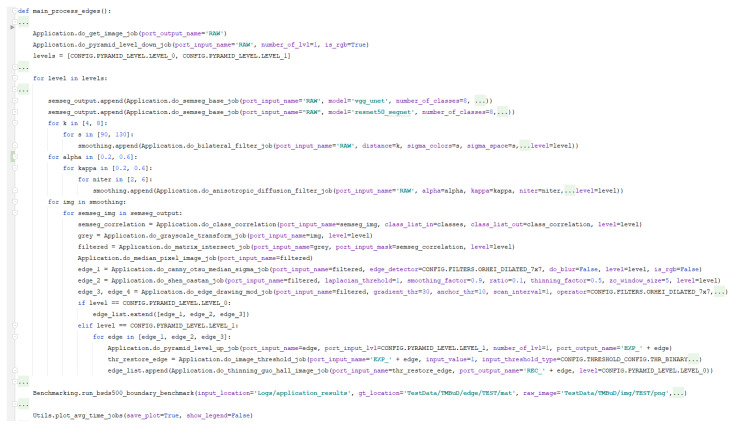
Setup in EECVF of the example.

The setup code corresponding to the application experiment is presented in Figure 20. As input, the system uses the test subset of TMBuD dataset that consists of 35 images of buildings. When adding the smoothing jobs to the pipelines we took in consideration several variations of configuration parameters. This causes the pipeline to create divergent paths that result in several new outputted edge maps at the end.

In Figure 20, one can see how we configured the CV pipeline. To make the user interaction easier with the framework, jobs return the output port names that they create. This is an important feature because we use the port names to link the flow of jobs.

Like stated in the introduction of the example, we smooth the input image with several variants of the Bilateral filter [54] and Anisotropic Diffusion filter [55]. This is done by re-triggering the job in the user–interface with different parameters. One important aspect when doing this is to change the name of the output port. Not changing the name will cause the job to be discarded as it will not bring added value to the final pipeline.

All the offered jobs from the framework have default output port names. This feature is added so that users do not need to add a specific name to the output port. As we can see in Figure 20, most of the jobs that we use do not configure the output name parameter and rely on the default names.

When configuring our edge detection jobs, do_canny_otsu_median_sigma_job for Canny [51] and do_edge_drawing_mod_job for the ED [53] are using the Orhei operator [60] that is dilated [61] with a factor of 2. For the Shen–Castan [52] operator, we are using the standard binary Laplace operator.

We observe from Figure 20 that the output ports from the edge detection jobs are being saved in a list. This list will be used later by us to specify to the configure_save_pictures service which ports we want to save. Afterwards, we will use the list to configure what jobs to evaluate using the Benchmarkingblock. In both cases, run_FOM_benchmark and run_bsds500_boundary_benchmark, we need to set the following parameters: location of output images, location of ground-truth images, location of original images and a list of ports to evaluate.

**Figure 21:**
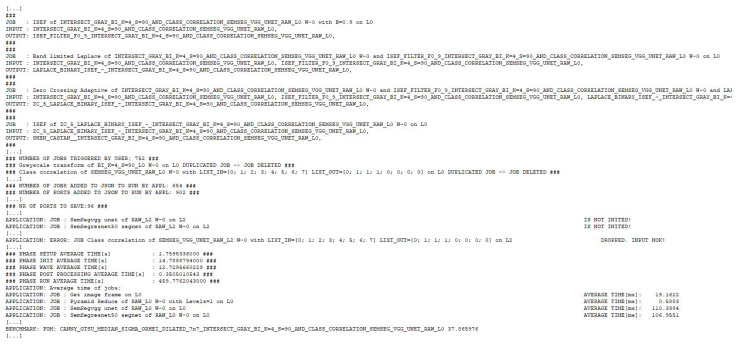
Console output of the application.

In Figure 21, we presented a part of the data that EECVF outputs when executed. First thing we can observe is the fact that the pipeline described is trimmed to the slimmest one possible, like stated in Algorithm 1. In this example, we triggered a number of 752 jobs that are parsed by the algorithm and in the end only 654 unique jobs with 902 ports are executed. For example, the Applicationblock handles the duplication of do_grayscale_transform_job. This job is triggered individually by us, but it is part of the edge detection jobs too.

As described in Section 3, the framework will add by itself jobs if they are needed. An example of this, we can see Figure 20 where the user triggered do_shen_castan_job but EECVF adds to the pipeline the following jobs, as we can see in Figure 21: do_isef_filter_job the specific ISEF filter, do_laplacian_from_img_diff_job or other Laplace variant configured by users, followed by do_zero_crossing_adaptive_window_isef_job, and do_threshold_hysteresis_isef_job as the last step of the algorithm. These jobs are not random but they are the inner steps of the Shen–Castan algorithm and we do this for a better optimization of jobs and ports. This aspect of jobs being broken up in smaller jobs is detailed in the description of each individual job.

We can observe in Figure 21 that the framework can handle missing data or corrupted flows. As we mentioned in the beginning of the example, we triggered a branch of the pipeline to run for the second pyramid level without the input image being there. The framework offers the information that the job was triggered by the user but could not run.

**Figure 22:**
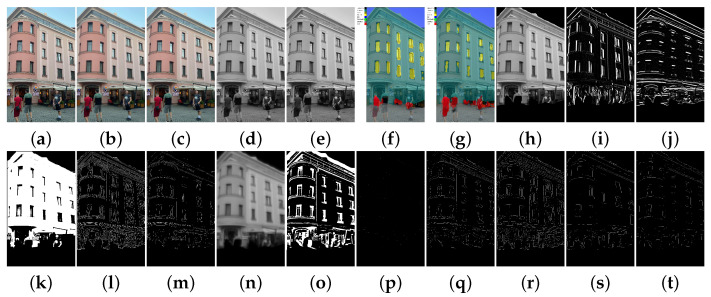
(**a**) Raw image; (**b**) Bilateral smoothing results; (**c**) Anisotropic smoothing results; (**d**) Gray transform of b; (**e**) Gray transform of c; (**f**) ResNet_SegNet result of a; (**g**) VGG_Unet result of a; (**h**) Intersection of f with c; (**i**) $G_{x}$ kernel of h. (**j**) $G_{y}$ kernel of h. (**k**) Otsu result of h; (**l**) Canny result of h; (**m**) ED result of h; (**n**) ISEF result of h; (**o**) Binary Laplace of h; (**p**) Zero-Crosing of o; (**q**) Shen-Castan of h; (**r**) Expanded Canny from L1; (**s**) Expanded ED from L1; (**t**) Expanded Shen-Castan from L1.

Some images from the processing of the pipeline are presented in Figure 22. This is done in order to demonstrate that the pipeline executed all the steps described in the example presented in Figure 10. The list of saved ports can be easily configured using the configure_save_pictures. This service will communicate to the Dataprocessingblock how to configure the saving of ports. This is an important aspect to configure because the number of ports to save will affect the run-time and the memory size that the application will take on the hardware. We could not show all the 96 edge maps that the pipeline exports at the end because of the lack of space in this paper, but the example can be reproduced from the EECVF repository.

**Figure 23:**
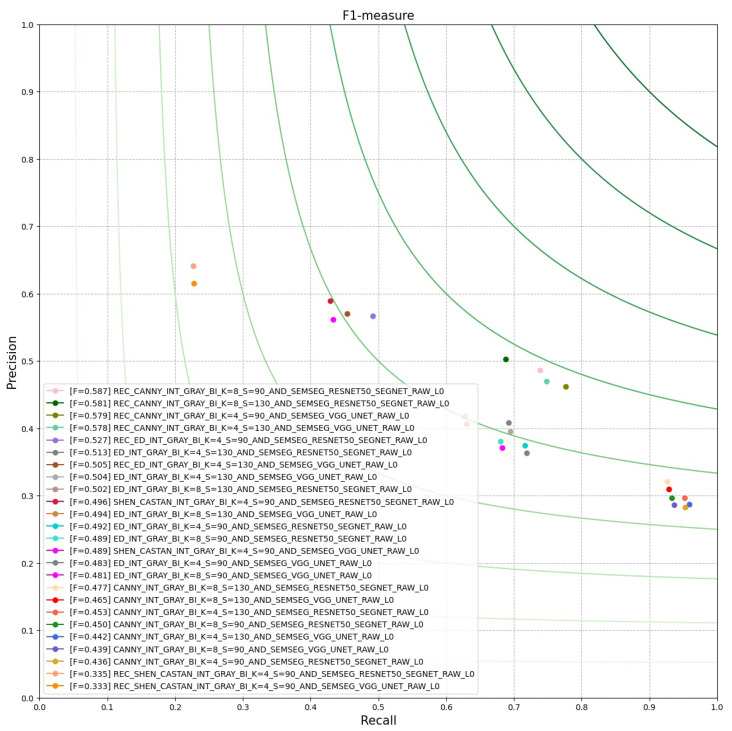
ROC plot of best 25 best PCM results.

**Figure 24:**
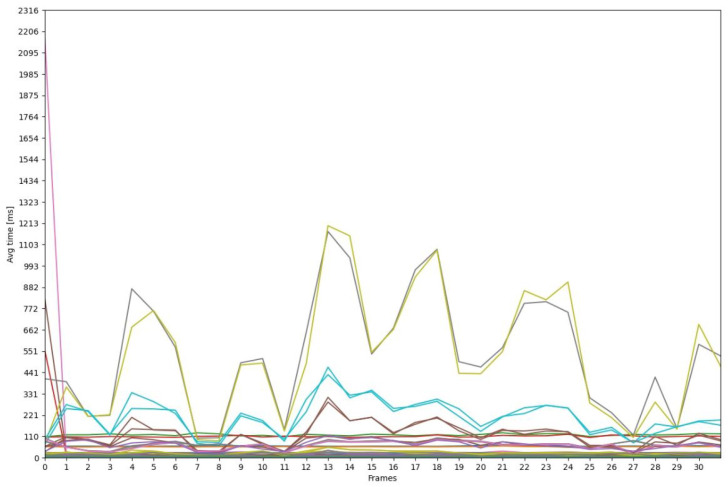
Average runtime of jobs.

For our experiment, we aim to evaluate our resulting edge-maps, using the Benchmarkblock. In Figure 23, we have plotted the best 25 results of PCM evaluation done by the run_bsds500_boundary_benchmarkjob. This is done by using the job from the DataProccessingblock: plot_first_cpm_results. In Figure 24, we have plotted the average run-time; this is a commonly desired information when executing a CV pipeline. Furthermore, we have configured plot_avg_time_jobs to not add any legend, because the pipeline consists of a high number of jobs.

In this section, by using the experimental pipeline, we have demonstrated that the proposed framework, EECVF, is capable of handling, configuring, and evaluating a complex CV pipeline with several paths and a big number of jobs and ports. Another important aspect to reiterate is the robust and stable error handling mechanism the framework has incorporated. Using parts of code that were presented in Figure 14 and Figure 20, we desired to give examples on how to configure such a pipeline. Users can execute the main_eecvf_jurnal.py example from the framework repository to reproduce the example.

## 5. EECVF Used in Education and Research

Teaching image processing techniques to students is often challenging. Image processing requires knowledge on the inner workings of the visual system of humans and on how they process the acquired visual information. It also requires that students understand how the visual effect of image processing relates to the mathematical algorithms used in processing.

In the past years, CV has touched upon all fields of modern life, including teaching and education. One of the common ways in which CV is involved in this field is through Augmented and Virtual Reality technologies [62,63,64,65,66,67]. If this is well understood by the students, they may proceed to the implementation stage of an image processing algorithm using a specific programming language [68].

A comprehensive review of computer vision education [69] points out the following areas in which EECVF might bring specific benefits:

**Usage in core courses**. Core courses in Computer Science are usually too oriented towards computers themselves and not towards real-world applications. Thus, lab exercises and homework assignments are not engaging enough for students to create positive learning. Introduction of Computer Vision examples and applications in courses such as programming, data structures, algorithms, math, or hardware can make the mostly theoretical knowledge more interesting and practical for students [69]. Basic algorithms can be demonstrated as images processing algorithms, data structures can be taught as inputs or outputs for a Computer Vision pipeline, programming assignments can be formulated so that they generate a processed image at the end, and so on.

**Effective and flexible software tool**. CV is a vast domain and students need guidance and predictability when tackling a CV assignment. Even a simple system is hard for them to implement in a short period of time if they need to develop everything from scratch [69].

**Tool for teachers to design assignments**. A source of difficulty in every CV course is the need to cover, in the same semester, both basic methods and algorithms and the latest findings and applications in the field. To do this, teachers need to be able to explain specific tasks in the CV process but also demonstrate real-world applications that ignite and maintain the interest of the students [69].

**Usage in research for education**. Research is insufficiently exploited and integrated in student education, as a means of enhancing their critical thinking skills, creativity, and ability to work in collaborative projects [69].

Although we have drawn some positive empirical insights from using the EECVF framework with our students, we plan to run a comprehensive research study to determine to what extent EECVF can alleviate the previous obstacles in computer vision education and research through the following features: breaking down a CV application in manageable steps; hiding the inner workings of a CV pipeline when this is not important for the course-specific task; out-of-the-box debugging capabilities.

## 6. Conclusions

EECVF is a open source Python-based framework that aims to assist researchers and teachers on day-to-day activities in the CV domain. As we presented in Section 3, the EECVF is a complex framework but facile to use. EECVF incorporates all the needed steps for a research activity in the CV domain.

Because of the configuration layer for each block, the user does not need to understand the way the predefined jobs are implemented. Another benefit is that users do not need to concern themselves with the data processing flow because that is embedded in the framework.

In Section 2, we presented a literature review and analysis of existing frameworks, highlighting the strong points of each. As we can observe, in recent years, the Python language has become the preferred programming language for the existing framework solutions. This trend is supported by Python’s capacity to interact with other programming languages and its cross-platform design.

We consider that, through the example we described in Section 4, we managed to present the “one click” quality of our framework. As we saw, users can configure mixed CV pipeline (classic and AI elements) upon the desired information and the results are saved and evaluated. This aspect is important when we consider that, in a modern CV application, data processing has become an essential process.

EECVF is a easy to use, modular and flexible software framework that can handle complicated CV pipelines while offering all the necessary information to the user for understanding the effect of each block in the chain. Combining the robust design of our framework with the advantages of Python programming language has proven to be beneficial to the outcome. EECVF can run in multiple operating systems with minimal changes.

For further work, we intend to focus on improving the inner workings of the framework and offer much more information regarding the resource and job management. We consider for this improvement to use external tools or libraries like SLURM [70].

The EECVF is in our perspective a continuously evolving framework. We believe that new image processing concepts, jobs, and services will be added due to day-to-day research or educational activities. The rate of EECVF development will probably be constant with the evolution of the CV domain.

## Data Availability

Not applicable.
